# The distribution and impact of viral lineages in domains of life

**DOI:** 10.3389/fmicb.2014.00194

**Published:** 2014-04-30

**Authors:** Arshan Nasir, Patrick Forterre, Kyung Mo Kim, Gustavo Caetano-Anollés

**Affiliations:** ^1^Evolutionary Bioinformatics Laboratory, Department of Crop Sciences, Illinois Informatics Institute, University of Illinois, Urbana-ChampaignUrbana, IL, USA; ^2^Unité BMGE, Institute PasteurParis, France; ^3^Institut de Génétique and Microbiologie, Université Paris-Sud, CNRS UMR8621Orsay, France; ^4^Microbial Resource Center, Korea Research Institute of Bioscience and BiotechnologyDaejeon, Korea

**Keywords:** viruses, evolution, replicon, capsids, virion morphotype, diversity, domains of life

Living organisms can be conveniently classified into three domains, Archaea, Bacteria, and Eukarya (Woese et al., 1990). The three domains are united by several features that support the common origin of life including the presence of ribosomes, double-stranded DNA genomes, a nearly universal genetic code, physical compartments (i.e., membranes), and the ability to carry out metabolism and oxidation-reduction reactions. In comparison, other types of genetic material and particles (e.g., viruses, plasmids, and other selfish genetic elements) are often excluded from the definition of “life” (for opposing views see Raoult and Forterre, 2008; Forterre, 2011, 2012a). However, they can still influence the evolution of cellular organisms, and in conjunction, establish complex life cycles.

Viruses impact our economy, medicine and agriculture due to their infectious nature. Viral infections transform the host cell into a virocell that no longer divides by binary fission but produces more viral particles or a ribovirocell in which the viral and cellular genomes coexist, the cell still dividing while producing virions (Forterre, 2011, 2012a). The virosphere (i.e., collection of all viruses) displays exceptional variability in virion morphologies and replication strategies. Viruses can be classified into DNA or RNA viruses, retroviruses or intermediate forms depending upon the type of replicon present inside the viral particle. Moreover, replicons could be linear, circular, single-stranded, double-stranded, or even segmented. The unprecedented diversity of replicon types has led to the proposal that viruses first invented DNA as means to trick the host defense systems (Forterre, 2002, 2005). Viruses can also transfer genes between species and enhance biodiversity (Nasir et al., 2012). Even more importantly, viruses appear to create massive amount of new genetic information, part of which can transfer to cells (Abroi and Gough, 2011; Forterre, 2011, 2012b). The discovery of “giant” viruses such as mimiviruses (La Scola et al., 2003), megaviruses (Arslan et al., 2011), pandoraviruses (Philippe et al., 2013), and pithoviruses (Legendre et al., 2014) now creates a continuum in genome size and functional complexity between the virosphere and cells. Still, viruses are neglected in phylogenetic studies because they lack a unifying genetic marker, similar to rRNA for cells, and because many biologists underestimate their genetic creativity. As a consequence, their role in the origin and evolution of modern life, and their impact on the ecology of our biosphere continue to be for the most part unrecognized (Koonin and Wolf, 2012). In this opinion article, we address the impact of viruses on the evolution of cells. We argue that viruses likely initiated major evolutionary shifts. Specifically, we consider that gain and loss of viral lineages often leads to divergent evolutionary trends even in closely related species. We emphasize that no evolutionary theory could be complete without accounting for the viral world and that viruses are responsible for ongoing adaptations in the cellular domains (see also Prangishvili et al., 2006; Forterre and Prangishvili, 2013; Koonin and Dolja, 2013).

The distribution of the association of viral replicon types with cells is extremely biased. For example, RNA viruses are completely absent in Archaea and are rare in Bacteria. In comparison, vertebrates host numerous RNA and retroviruses. Surprisingly, dsDNA viruses are rare in plants while dsRNA viruses are abundant in fungi. Similarly, retroviruses are integrated into the genomes of multicellular eukaryotes but are completely absent in the microbial genomes. In other words, specific relationships exist between the type of viral replicon and the host range. Viruses with a particular replicon may infect one group of organisms but may not replicate in another. Big jumps of viruses from one cellular lineage to another have been observed within the eukaryotic “division” such as animals (opisthokonts) and plants (viridiplantae), when a virus adapts to an established consortium of ecological partners. The same virus can sometimes infect both plant and animal cells when these are linked by their mode of life. One example is the Fiji disease virus (*Reoviridae*) that can replicate in both its insect vector (Delphacidae) and flowering plants (Kings et al., 2012). However, no modern virus is known to cross the barrier between domains. Therefore, while viruses may be able to jump hosts over short evolutionary time spans, crossing domain boundaries is less likely and not expected to compromise our inferences.

To obtain a quantitative view of viral diversity and its distribution among cellular domains, we extracted genome data from the Viral Genomes Resource at NCBI (Bao et al., 2004). This resource provides accurate, manually curated information about sequenced viral genomes that is minimally redundant. Generally, one sequenced genome portrays many isolates/strains of the same virus. Specifically, we investigated the host preferences for viruses with different replication strategies (Figure 1A) and contrasted virion morphologies (borrowed from ViralZone; Hulo et al., 2011) of virus families infecting different domain groups (Figure 1B). A birds-eye view of the distribution of viruses among hosts revealed that only 63 were exclusive to the archaeal domain (hereinafter referred to as archaeoviruses) (Figure 1A). In comparison, 1251 bacterial (bacterioviruses, formerly bacteriophages) and 2321 eukaryal viruses (eukaryoviruses) were identified. The low number of archaeoviruses is clearly due to a sampling bias (e.g., the low number of archaeal species screened for the presence of viral infection) since it has been shown that four different viruses can infect a single archaeal species (i.e., *Aeropyrum pernix*), each from a different family (Mochizuki et al., 2010, 2011, 2012). Despite their low number, archaeoviruses exhibit greater virion morphotype diversity compared to bacterioviruses [e.g., 4 unique virion morphotypes vs. none (Figure 1B); see also Pietilä et al., 2014]. In comparison, bacterial organisms host a vast number of described DNA viruses (1178 out of total 1760) but display very little family and morphotype diversity. In fact, 95% of the dsDNA bacterioviruses belong to just one order (Caudovirales) and only three families (*Myoviridae*, *Podoviridae*, and *Siphoviridae*). Moreover, only 9 virion morphologies have been observed in bacterioviruses (compared to 16 in archaeoviruses) (Pietilä et al., 2014). One explanation for the low diversity of bacterioviruses could be the invention of peptidoglycan-containing cell wall in Bacteria. The inability to traverse this barrier likely resulted in loss of many viral lineages in Bacteria (Forterre and Prangishvili, 2013; Prangishvili, 2013). Taken together, these observations suggest that Archaea are likely infected by a greater number of viral lineages than Bacteria. This is showcased by their virion morphologies diversity Figure 1) (Pina et al., 2011; Pietilä et al., 2014), (which is expected to grow with improvements in our ability to isolate viruses from atypical habitats.

**Figure 1:**
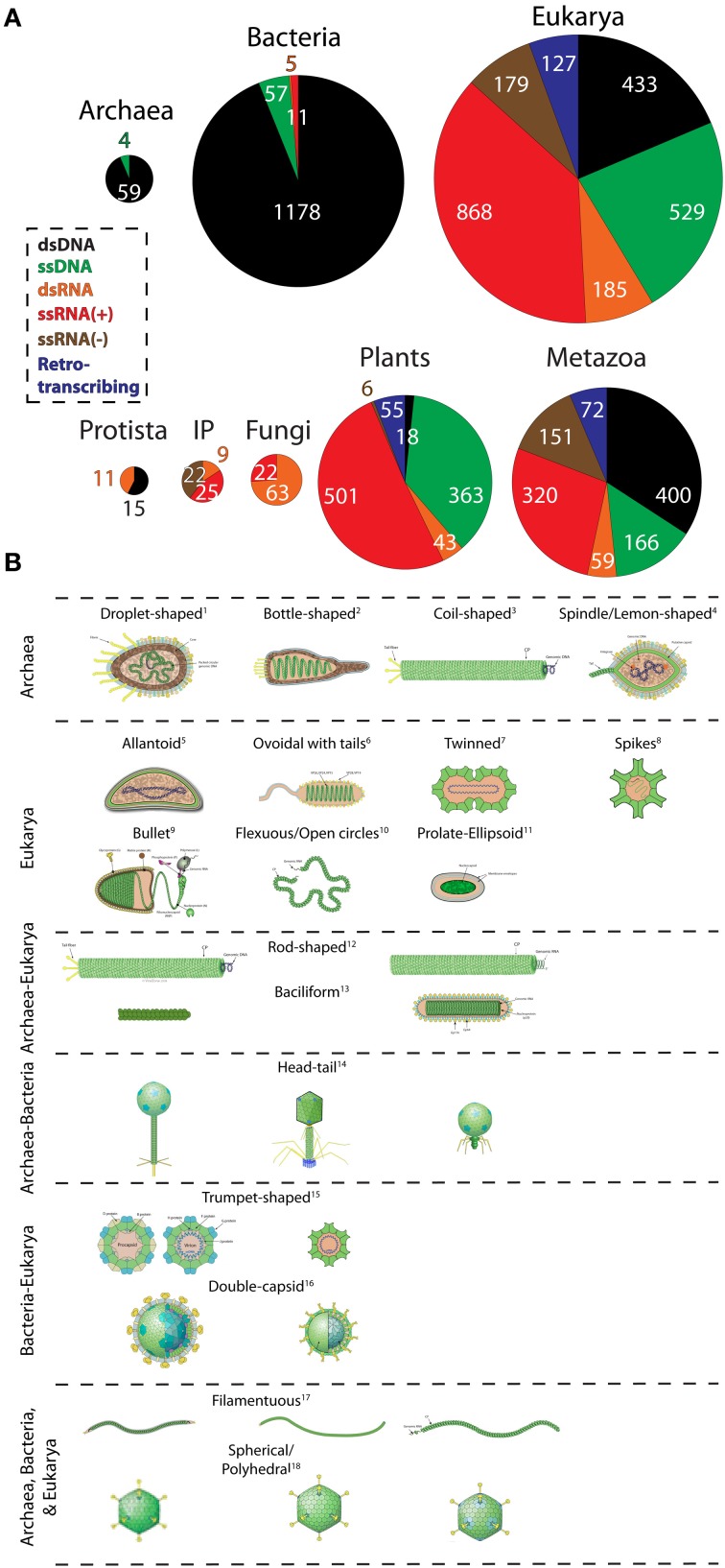
**The abundance and diversity of viral lineages in the domains of life. (A)** Pie-charts describe the abundance of dsDNA, ssDNA, dsRNA, ssRNA(+), ssRNA(−), and retrotranscribing viruses in Archaea, Bacteria, and Eukarya, and within the major eukaryal divisions. Genome data from 3660 completely sequenced viral genomes corresponding to 1671 dsDNA, 610 ssDNA, 883 ssRNA(+), 179 ssRNA(−), 190 dsRNA, and 127 retrotranscribing viruses were retrieved from the Viral Genomes Resource (April, 2014). Additionally, two ssDNA archaeal viruses were identified from the literature (Pietilä et al., 2009; Mochizuki et al., 2012). Viruses that were unassigned to any order, genera, or species and unclassified viruses were excluded from sampling. Viruses were broadly classified according to host preferences into the following categories: Archaea, Bacteria, Protista (animal-like protists and brown algae), Invertebrates and plants (IP); Fungi (all fungi and fungi-like protists); Plants (all plants, green algae, and diatoms), and Metazoa (vertebrates, invertebrates, and human). Host information was available for roughly 99% (3633) of the sampled viruses. Pie-charts are proportional to the size of each distribution. **(B)** Virion morphotypes that are specific to a domain or are shared between domains are displayed. Virion pictures were borrowed from the ViralZone web-resource (Hulo et al., 2011) and from Pietilä et al. (2014) and Pina et al. (2011). A keyword-based search was performed on text data to assign the most general morphotypes (e.g., rod-shaped, spherical, droplet-shaped, etc) to all viruses. More than one viridae with same morphotype is possible but not made explicit. The diagram does not always imply evolutionary relationship between viruses harboring common morphology. For example, archaeal and eukaryal rod-shaped viruses are probably not evolutionarily related (Goulet et al., 2009). Well-studied exceptions are head-tail caudovirales harboring the HK97 capsid fold and of polyhedral viruses harboring the double jelly roll fold (Abrescia et al., 2012). ^1^*Guttaviridae*; ^2^*Ampullaviridae*; ^3^*Spiraviridae* [name pending approval by ICTV]; ^4^*Fuselloviridae*; ^5^*Ascoviridae*; ^6^*Nimaviridae*; ^7^*Geminiviridae*; ^8^*Astroviridae*; ^9^*Rhabdoviridae*;^10^*Ophioviridae*; ^11^*Polydnaviridae*; (left to right) ^12^*Rudiviridae* (Archaea); *Virgaviridae* (Eukarya); ^13^*Clavaviridae* (Archaea) *Roniviridae* (Eukarya); ^14^*Siphoviridae, Myoviridae*, and *Podoviridae* (Archaea and Bacteria); ^15^*Microviridae* (Bacteria), *Circoviridae* (Eukarya); ^16^*Cystoviridae* (Bacteria), *Reoviridae* (Eukarya); ^17^*Lipothrixiviridae* (Archaea), *Inoviridae* (Bacteria), *Potyviridae* (Eukarya); ^18^*Sulfolobus* turreted icosahedral virus (Archaea), *Tectiviridae* (Bacteria), *Adenoviridae* (Eukarya).

Interestingly, all archaeoviruses possess DNA replicons but no RNA genomes. The complete absence of RNA viruses in Archaea can be linked to high temperature RNA instability (Forterre, 2013). We speculate that escape from RNA viruses could be one major trigger for the evolution of modern Archaea (Forterre, 2013). Thus, loss of RNA viral lineages likely initiated archaeal migration to the harsh environments. One recent study reported the isolation of ssRNA(+) viruses from an archaea-rich community in a hot, acidic spring of Yellowstone National Park (Bolduc et al., 2012). However, their host tropism could not be established with confidence. Finally, four ssDNA viruses were recently isolated from Archaea (Pietilä et al., 2009; Mochizuki et al., 2012; Sencilo et al., 2012). Of these, *Aeropyrum* coil-shaped virus (*Spiraviridae*) is the largest known ssDNA virus and displays unique coil-shaped virion morphology (Figure 1B; Mochizuki et al., 2012).

Bacterioviruses are remarkably successful in Bacteria and are highly abundant. Their virions outnumber their bacterial hosts in oceans, balance microbial populations in the marine communities, and regulate biogeochemical cycles (Breitbart and Rohwer, 2005; Suttle, 2007; Rohwer and Thurber, 2009; Zhao et al., 2013). Among the dsDNA bacterioviruses, tailed-bacteriophages exhibit extensive similarities with archaeal caudovirales, suggesting that they form a monophyletic group (Krupovic et al., 2010). Archaeal and bacterial caudovirales have indeed been grouped in a single major evolutionary lineage, together with *Herpesviridae*. All of these viruses share the same Hong Kong fold (HK97) in their major capsid proteins and homologous packaging ATPases (Baker et al., 2005; Pell et al., 2009; Krupovic et al., 2010; Abrescia et al., 2012). Notably, it has been found recently that the capsid of *Herpesviridae* exhibits a small tail similar to those of *Podoviridae* (Schmid et al., 2012). These data suggest that viruses of the HK97-like lineage are very ancient and originated (most likely) prior to the last common ancestor of cells. Another example of viral lineage shared by the three domains is the so-called “PRD1/Adenovirus lineage” of dsDNA viruses characterized by a major capsid protein containing the double-jelly roll fold and a common packaging ATPase (Abrescia et al., 2012). In comparison, ssDNA bacterioviruses are not as successful in Bacteria and correspond to two major families, *Inoviridae* and *Microviridae* (smallest genomes among DNA viruses; Rosario et al., 2012). Viruses in this group replicate by converting their single-stranded DNA genome into a double-stranded intermediate form engineered by host polymerase. These viruses lack their own polymerase and share this property with the ssDNA viruses of Archaea and Eukarya.

In contrast to DNA viruses, RNA viruses are not as successful in Bacteria. Only, 5 dsRNA, and 11 ssRNA(+) bacterioviruses could be identified. In turn, none of the ssRNA(−) and retrotranscribing viruses associated with bacterial hosts. Among the RNA bacterioviruses, dsRNA viruses (*Cystoviridae*) encode segmented genomes and infect mostly *Pseudomonas* species (Silander et al., 2005). Interestingly, *Cystoviridae* closely resembles eukaryal dsRNA viruses (i.e., *Reoviridae* and *Totiviridae*) in terms of life cycle and homologous RNA-dependent-RNA-polymerase gene sequences (a virus hallmark) (Butcher et al., 1997). Unlike Archaea, Bacteria are also infected by ssRNA(+) viruses (*Leviviridae*). These viruses are amongst the simplest and smallest known viruses, and historically yielded useful insights into mRNA function (Bollback and Huelsenbeck, 2001). Because RNA viruses (ssRNA and dsRNA) infect both Bacteria and Eukarya, their ancestors likely originated from a putative ancient world of cells with RNA genomes and RNA viruses (Forterre, 2005, 2006a,b). This points to the ancient existence of RNA viruses and suggests their loss from Archaea (since loss in one domain is more likely than the independent gain in two!). The instability of RNA at high temperatures supports this hypothesis, since it is likely that the last common ancestor of Archaea was a hyperthermophile (Brochier-Armanet et al., 2011).

Viruses with all possible types of replicons infect eukaryal organisms. RNA viruses are predominant and cover the entire taxonomic range within Eukarya (Figure 1A). Eukaryoviruses also exhibit many unique virion morphotypes not observed in the prokaryotic viruses and are unequally distributed in the major eukaryal groups (Figure 1). For example, dsDNA viruses are completely absent in fungi and are rare in plants (i.e., only found in green algae). This suggests that these groups have evolved sophisticated mechanisms to eliminate dsDNA viral infections. A good candidate is the cell wall structure found in plants, fungi, and algae. Differences in cell wall composition and rigidity greatly limit means of viral entry into the cell and serve as barriers to viral infections (Dimmock et al., 2007). However, loss of one viral lineage is apparently offset by the gain of other lineages. This is evident from the high RNA virus distribution among plants and fungi. The origin of the diversity and abundance of RNA viruses in eukaryotes but their near absence in prokaryotes is particularly puzzling (Koonin et al., 2006). For example, ssRNA(−) and retroviruses are highly successful in vertebrates. At first glance, it seems that organism complexity is proportional to the variety of viral infections. For instance, metazoa are infected by a host of retroviruses. Retroviruses can integrate their genomes into host DNA and thus alter gene expression patterns and trigger genomic rearrangements (Arkhipova et al., 2012). These activities can lead to production of novel genes and advanced machineries (Forterre, 2013). In fact, telomerase enzymes are homologous to retroviral proteins and neocentromeres are formed by epigenetic regulation of transposable elements (Singer, 1995; Chueh et al., 2009), both likely transferred from viruses to host cells much earlier in evolution. This argument is further supported by the absence of RNA and retroviruses from unicellular eukaryotes such as yeast, which resemble a prokaryotic lifestyle (Forterre, 2013). Thus, co-evolution between viruses and their hosts may have led to organism complexity in the eukaryotic domain.

The diversity of eukaryoviruses is intriguing, both in terms of genome structure and virion morphology (see Figure 1B). In particular, retrotranscribing, ssRNA(−), and many DNA virus families are only present in eukaryotes. Surprisingly, although Archaea and eukaryotes are very similar in term of their basic molecular biology, there are no viral lineages specific for these two domains (Forterre, 2013). Virions with rod-shaped morphology are up to now specific for Archaea and Eukarya (Figure 1B), but they harbor DNA and RNA genomes, respectively, and it is unclear if their major coat proteins are evolutionary related (Goulet et al., 2009). The same is probably also true for bacilliform viruses. Notably, the diversity and specificity of eukaryoviruses is difficult to reconcile with the archaeon-bacterium fusion scenarios for the origin of eukaryotes (e.g., Martin and Müller, 1998), as recently argued (Forterre, 2013).

To conclude, the distribution of viral lineages follows an ancient, highly dynamic and ongoing process that impacts the evolution of organisms. New viral lineages often arise from existing ones and may cross species barriers to infect new hosts (e.g., parvovirus; Shackelton et al., 2005), putting enormous evolutionary pressure on cellular organisms and prompting them to unfold molecular and cellular innovation (Forterre and Prangishvili, 2009) in the search of either simplicity or complexity.

## Conflict of interest statement

The authors declare that the research was conducted in the absence of any commercial or financial relationships that could be construed as a potential conflict of interest.
